# A Novel Method for High Temperature Fatigue Testing of Nickel Superalloy Turbine Blades with Additional NDT Diagnostics

**DOI:** 10.3390/ma14061392

**Published:** 2021-03-12

**Authors:** Dominik Kukla, Mateusz Kopec, Ryszard Sitek, Aleksander Olejnik, Stanisław Kachel, Łukasz Kiszkowiak

**Affiliations:** 1 Institute of Fundamental Technological Research, Polish Academy of Sciences, Pawińskiego 5B, 02-106 Warsaw, Poland; dkukla@ippt.pan.pl (D.K.); mkopec@ippt.pan.pl (M.K.); 2 Department of Mechanical Engineering, Imperial College London, London SW7 2AZ, UK; 3 Faculty of Materials Science and Engineering, Warsaw University of Technology, Wołoska 141, 02-507 Warsaw, Poland; ryszard.sitek@pw.edu.pl; 4 Faculty of Mechatronics Armament and Aerospace, Military University of Technology, 2 Gen. Sylwestra Kaliskiego Str., 00-908 Warsaw, Poland; aleksander.olejnik@wat.edu.pl (A.O.); stanislaw.kachel@wat.edu.pl (S.K.)

**Keywords:** nickel superalloys, high temperature fatigue, finite element (FE) modelling, eddy current

## Abstract

In this paper, a novel method for high temperature fatigue strength assessment of nickel superalloy turbine blades after operation at different times (303 and 473 h) was presented. The studies included destructive testing (fatigue testing at temperature 950 °C under cyclic bending load), non-destructive testing (Fluorescent Penetrant Inspection and Eddy Current method), and finite element modelling. High temperature fatigue tests were performed within load range from 5200 to 6600 N using a special self-designed blade grip attached to the conventional testing machine. The experimental results were compared with the finite element model generated from the ANSYS software. It was found that failure of turbine blades occurred in the area with the highest stress concertation, which was accurately predicted by the finite element (FE) model.

## 1. Introduction

The power and efficiency of an aircraft engine mainly depends on the inlet gas temperature. Therefore, it is desirable to increase the temperature of the flue gas to the combustion temperature of aviation fuel, which is about 2300 °C. However, the limitations are the strength properties of the alloys from which the blades are made. Nickel super alloys are typically used in such conditions. Additional application of thermal barrier coatings (TBC) on nickel-based superalloys allows to increase the effective service temperature to 1300 °C, while the temperature on the blade attachment does not exceed 300 °C [1]. An aggressive environment is another factor that affects the durability of engine turbine blades. Fuel combustion products (e.g., Na_2_SO_4_, NaCl, V_2_O_5_), oxidation, hot corrosion, erosion, and foreign object damage could significantly reduce their service life [2]. Simultaneously, the aircraft engine turbine blades are subjected to high mechanical loads, both static and dynamic. These mechanical loads included:

- Loads caused by static and dynamic action of the flowing medium on the profile part of the blade,
- Mass loads caused by centrifugal forces,
- Loads caused by elastic vibrations of the blades and the entire rotor.

On the other hand, in the axial compressors in turbine blades the following stresses may occur:

- Tensile caused by centrifugal forces of the rotating masses of the shoulder blade and bandage,
- Bending from the action of the pressure of the medium on the profile part, from the centrifugal forces of the rotating blade mass, caused by transverse vibrations of the blade,
- Tangents from the action of the torsional moments of forces caused by the action of the flowing medium, from the action of the torsional moments of the blade mass forces, from the torsional vibrations of the blade’s working part,
- Normal in any blade cross-section, as the sum of normal component stresses.

It was also reported, that the vibration may affect the service life of turbine blades as they introduce cyclic loads into blade, causing its failure due to fatigue [3]. Therefore, it is necessary to monitor the structural condition of the turbine blades, especially in the area of the leading edge. Visual methods of gas turbine blades condition inspection include comparative analysis of the surface images of the tested blades with reference surfaces. Such a method is therefore very subjective as its depends on the diagnostician’s knowledge and experience [4]. The optical methods also include metallographic investigations on cross-sections or fracture surfaces of turbine blades after a long time of operation [5]. However, it should be mentioned that the quality of blades after production and renovation is often the main cause of their accelerated fatigue damage, thus the effectiveness of optical methods is limited [6,7]. Increasing demands of the aviation industry led to the development of new diagnostic techniques, that allow to identify surface cracks, intrinsic defects, subsurface defects, pores, and potential areas of crack initiation. These techniques include: blade-surface images analysis [8], optoelectronic and thermographic methods [9], eddy current and ultrasonic methods [10], vibrothermography [11], or even digital image correlation [12].

The mechanical properties and service life of turbine blades were mostly evaluated using standard specimens [12,13,14,15,16] and numerical stress-strain analysis or model predictions [2,17,18,19]. However, the results obtained from standard specimen testing are different from these obtained from the full-scale turbine blade testing due to its structural complexity [20]. Thus, new methods and experiments were developed to assess the fatigue response of nickel-based superalloy turbine blades. Beghini et al. [21] introduced a test rig, which allows us to reproduce the stress and strain cycle occurred in the fillet region between the trailing edge and platform of Gas Turbine (GT) cooled blades. Wang et al. [22] developed a complex thermomechanical fatigue (TMF) test rig with loading, heating, cooling, and control subsystems, in which a mechanical load of the test section was adjusted discretely by changing the combination of spacers in the joints. These methods allow us to understand the material behavior in particular regions of the real components, which leads to a better estimation of their service life.

However, in this paper, the simplest method for the high temperature fatigue testing of turbine blades was presented. The special turbine grip designed by authors allows us to introduce the cyclic loading to turbine blade surface using a standard testing machine. The geometry of such a grip enables high temperature fatigue testing of a full-scale turbine blade. The experimental results were compared with a finite element model of turbine performance generated from the ANSYS software. An additional non-destructive approach was made to identify the surface defects (fluorescent penetrant inspection) and the potential areas of crack propagation (eddy current method).

## 2. Materials and Methods

**Figure 1:**
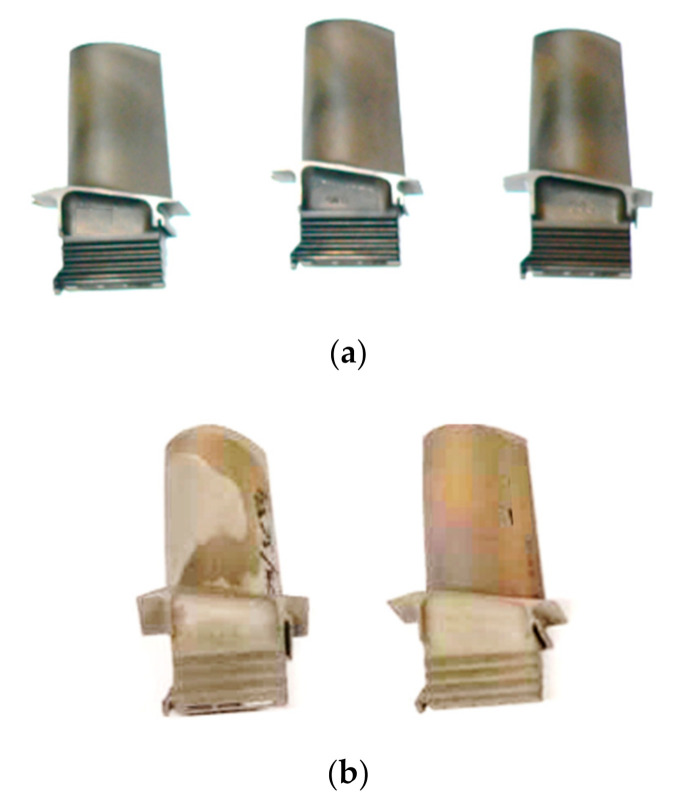
Turbine blades after exploitation for 303 h (**a**) and 473 h (**b**).

Ten turbine blades made of nickel superalloy operated during 303 h and 473 h (5 pieces each) provided by the aviation sector were subjected to mechanical and non-destructive testing. The exemplary turbine blades and their chemical composition are presented in Figure 1 and Table 1, respectively.

**Figure 2:**
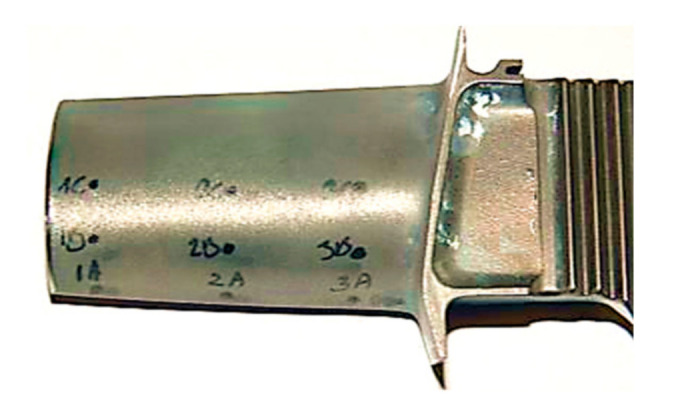
Places of impedance phase angle measurement.

The non-destructive testing included fluorescent penetrant inspection (FPI) and the eddy current (ET) method. The FPI tests were carried out in accordance with Polish standard PN-EN ISO 3452-1 [23] using Magnaflux sprays (Magnaflux Limited, Illinois Tool Works, Glenview, IL, USA) and an ultraviolet lamp. The ET tests were performed using a Olympus Nortec 600 D (Olympus, Tokio, Japan) flaw detector and pencil probes with operating frequencies of 100–500 kHz and 1–6 MHz. The signal was calibrated using a pattern sample with referenced electric discharge machined (EDM) notches of 0.1, 0.2, 0.5, and 1 mm depth. Such a sample allowed us to optimize the measuring parameters for the best detection of surface and subsurface defects. The eddy current method also enabled us to identify places with changes, due to extensive operating conditions, and properties. Both blade surfaces and leading edges were scanned to detect the areas of potential crack propagation as shown in Figure 2. However, it should be mentioned that the scanning of the leading edge was associated with a strong edge effect, which reduced the ability to detect cracks in this particular area.

**Figure 3:**
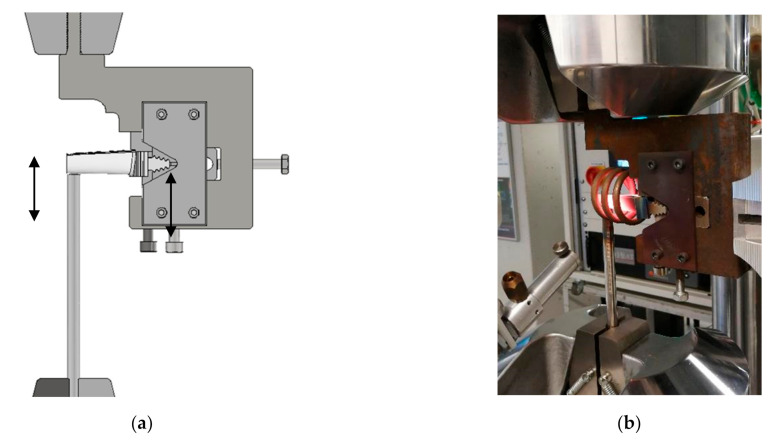
Schematics of a turbine blade grip (**a**) and its general view during the experiment (**b**).

Fatigue tests were performed on the MTS 810 testing machine (MTS System, Eden Prairie, MN, USA), with an axial force range of +/−250 kN, equipped with a FLEX digital controller (MTS System, Eden Prairie, MN, USA). The turbine blades were attached in the grip and heated with an induction heater (Ameritherm, New York, NY, USA) to a temperature of 950 °C. The load was implemented to the surface of the turbine blade in force control mode. Schematics of a turbine blade grip are presented in Figure 3a, while an experimental setup during fatigue test at 950 °C is presented in Figure 3b.

The range of fatigue loads was established on the basis of the force/displacement curve determined from the bending test at 950 °C. Fatigue tests were performed in the load amplitude range from 5200 to 6600 N. During each test, fatigue hysteresis loops were recorded in sequences every 50 cycles. The load was carried out at a frequency of 10 Hz, from 200 N to 5.2–6.6 kN in order to reduce the load impact of the shaft on the blade surface. Temperature stability was controlled using a bicolor infrared pyrometer, shown in Figure 3b. Fatigue tests were initiated after 1 h of heating the turbine blade, which was in contact with the loading rod. Such an arrangement allowed us to reduce the effect of thermal expansion on material behavior during testing. The results were presented in the form of fatigue hysteresis loops in selected load cycles.

Microstructural characterization and chemical composition analysis of the turbine blades were examined using Hitachi 2600 N scanning electron microscope with energy dispersive spectroscopy (EDS) attachment (Oxford Instruments, Oxford, UK).

## 3. Results and Discussion

### 3.1. Fluorescent Penetrant Inspection (FPI)

**Figure 4:**
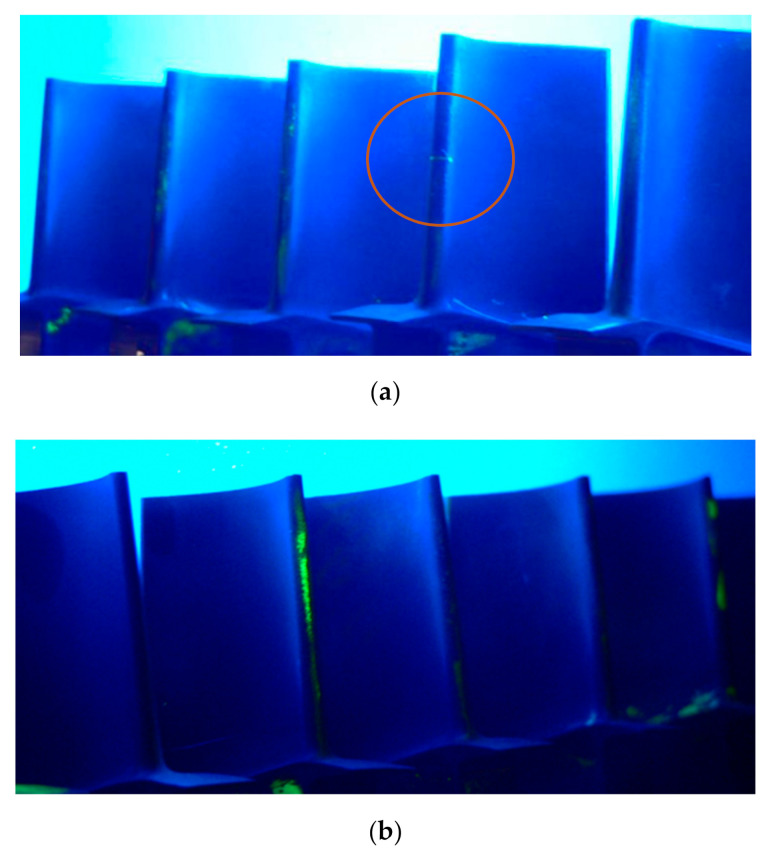
Fluorescent penetrant inspection (FPI) of turbine blades for series 1 (one crack) (**a**) and series 2 (no crack) (**b**).

Fluorescent penetrant inspection (FPI) is known as “dye penetrant inspection”. During such inspection, a fluorescent dye was applied to the surface of the object to detect structural defects that may affect the integrity or quality of the part in question. The yellow glow of the fluorescent penetrant caused by its reaction with ultraviolet light allows the FPI dye to sharply contrast with the dark background and further detect structural defects. FPI revealed high porosity at the leading edge of almost all blades of both series, as shown in Figure 4. Such porosity was probably caused by the operation under cyclic loads in a hot gas stream. Penetrant tests showed no cracks on the tested blades, with the exception of the one sample. Fluorescent indication of a crack on the turbine blade is marked in Figure 4a, where turbine blades are shown in UV light. The fracture was located approximately in the middle of the blade’s edge, oriented perpendicularly to the leading edge.

### 3.2. Eddy Current (ET)

Eddy current testing is an effective, non-destructive method that uses the electromagnetic induction to detect structural defects in conductive materials. The eddy current phenomenon is observed when an alternating current (AC) is applied via a probe or coil on the surface of the component in question. A magnetic field induced in the test piece allows the eddy current to flow. An immediate change of ET indicates the defects or discontinuities near the surface.

**Figure 5:**
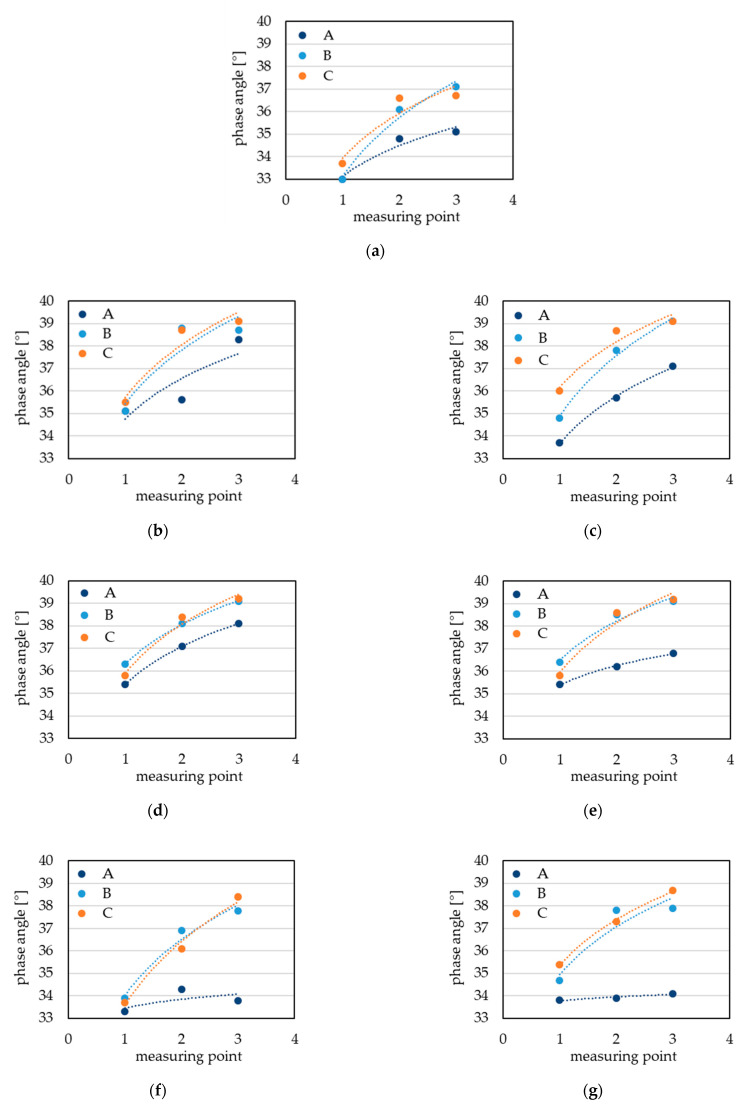
Phase angle measurements for different measuring points of turbine blades: as received state (**a**) after operation time of 303 h (**b**–**d**) and 473 h (**e**–**g**).

In this study, ET measurements were performed at three different locations near the blade’s edge (A) and 10 mm and 20 mm from the edge, as shown in Figure 2. The crack identified by the penetration method was not disclosed by the eddy current method due to its location at the edge of the blade. In this location, a strong edge effect occurred, which distorted the signal significantly. The high porosity of this area also affected the quality of the signal. The lowest values of the phase angle were registered at the top edge of the blade. Such a location is mainly subjected to extensive performance at a high temperature and thus the phase angle values were the lowest. However, it should be mentioned that the initial thickness of blade itself also affects the phase angle recordings [24]. Based on the phase angle measurements and their distribution shown in Figure 5, it could be concluded that the values obtained for the A regions clearly indicate the difference in operation time and simultaneously in material integrity for both types of turbine blade in question.

### 3.3. Numerical Strength Analysis of High Pressure Turbine Blade Performance

The numerical strength analysis of a high pressure turbine blade was performed using the finite element method (FEM) implemented in specialized software ANSYS, which is one of the most recognized codes for solving various types of problems in mechanics, especially in the field of strength analysis or fluid flow [25,26]. Moreover, the FEM is one of the most commonly used methods for solving engineering problems in the field of strength analysis. It is based on the discretization of a continuous model with infinite degrees of freedom into a system with finite degrees of freedom. After discretization, the divided area, which is the geometry of the research object, is represented by simple elements, such as triangles and squares. This means that even very complex structures can be analyzed. For the obtained elements, called finite elements, local approximation is made by functions called shape functions. The effect of discretization is the change of the problem of minimizing a function to the problem of minimizing the function of many variables. It is assumed that the value of a search function within the finite element can be determined from the value of the function at the nodes of the element. The division into finite elements is such that the elements have common nodes, which means that the continuity of the examined function between two elements is maintained.

**Figure 6:**
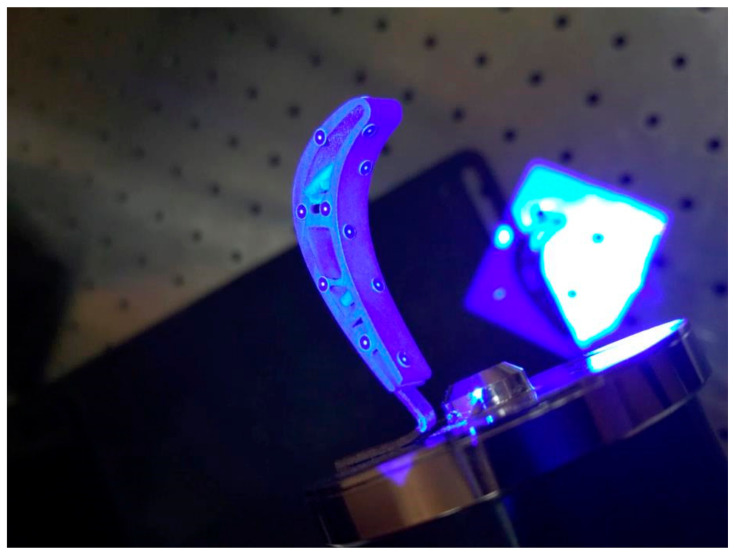
The turbine blade section during the digitization process.

**Figure 7:**
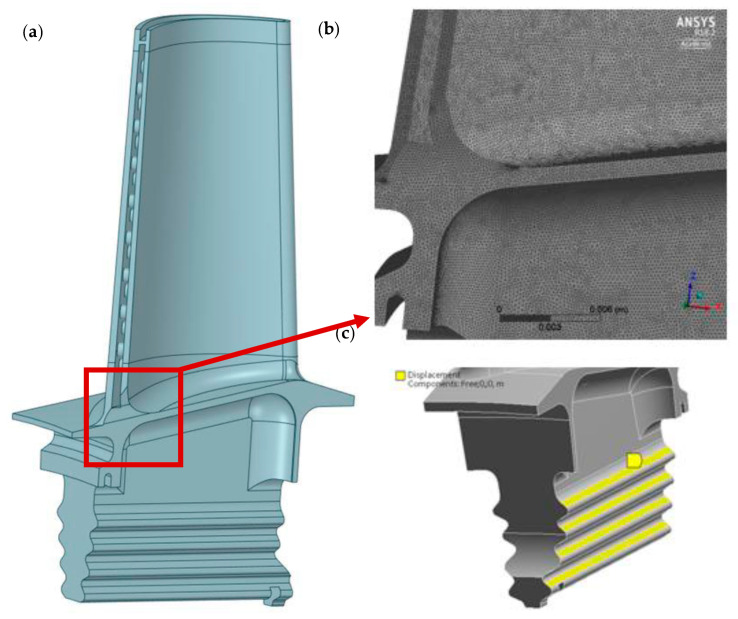
Created 3D CAD model of a high-pressure turbine blade (**a**) with computational mesh density (**b**); fix Boundary Condition on upper surfaces of the blade attachment (**c**).

The geometry of the high pressure turbine blade was developed as a result of the digitization process of outer and inner surfaces of the blade carried out by the Military University of Technology research team. The results of this process is usually the set of points (point cloud) that defines external geometry of an object. Thanks to the use of specialized research equipment and research staff with extensive experience in the field of reverse engineering, a reliable geometric CAD model was obtained, characterized by high accuracy of mapping surfaces. In order to create the geometry of the inner cooling channels, it was decided to cut the existing blade and make 3D scans of individual sections of the blade. Figure 6 shows one of the turbine blade sections during the digitization process. Furthermore, Figure 7 shows the created 3D CAD model of a high pressure turbine (HPT) blade.

In order to prepare the computational model of the turbine blade for strength analysis, we had to introduce appropriate corrections in the obtained surface geometry, enabling the generation of the computational mesh. A discrete model was obtained, consisting of about 9.5 mln nodes and 6.5 mln elements.

To perform numerical strength analysis, the following parameters were taken and assumptions were made:

− Blade was fixed on upper surfaces of the blade attachment, as shown in Figure 7c,
− Angular velocity: 1627 [rad/s],
− HPT inlet pressure: 2,021,434 [Pa],
− HPT outlet pressure: 966,777 [Pa],
− HPT blade mass: 0.139 [kg],
− Density of HPT blade material: 8320 [kg/m3],
− Temperature: 950 [°C],
− Elastic modulus of HPT blade material: 190 [GPa],
− Poisson ratio of HPT blade material: 0.3.

**Figure 8:**
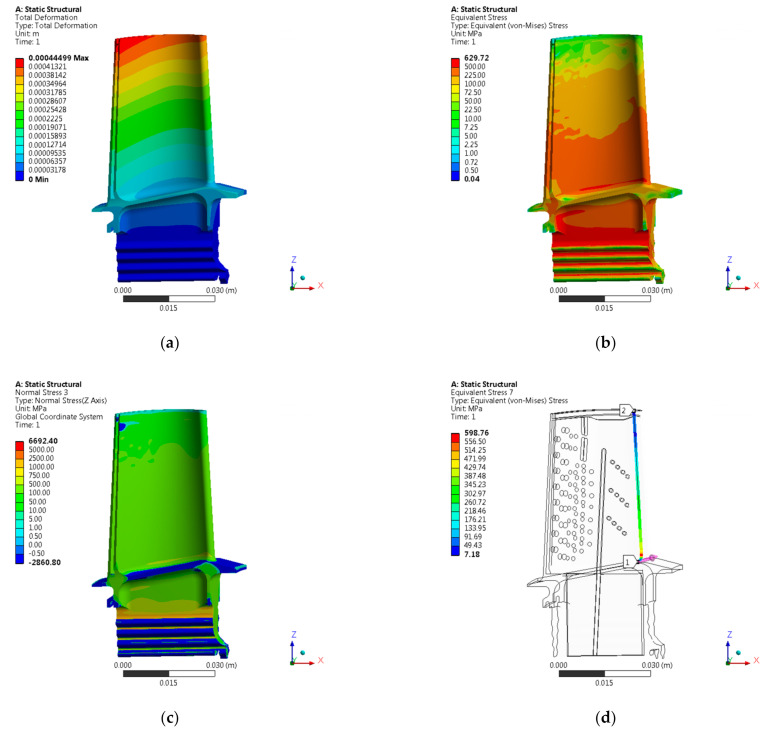
Results of HPT blade numerical analysis: (**a**) displacements [m], (**b**) equivalent stresses (von Mises) [MPa], (**c**) radial stresses [MPa], (**d**) equivalent stresses (von Mises) on blade leading edge [MPa].

**Figure 9:**
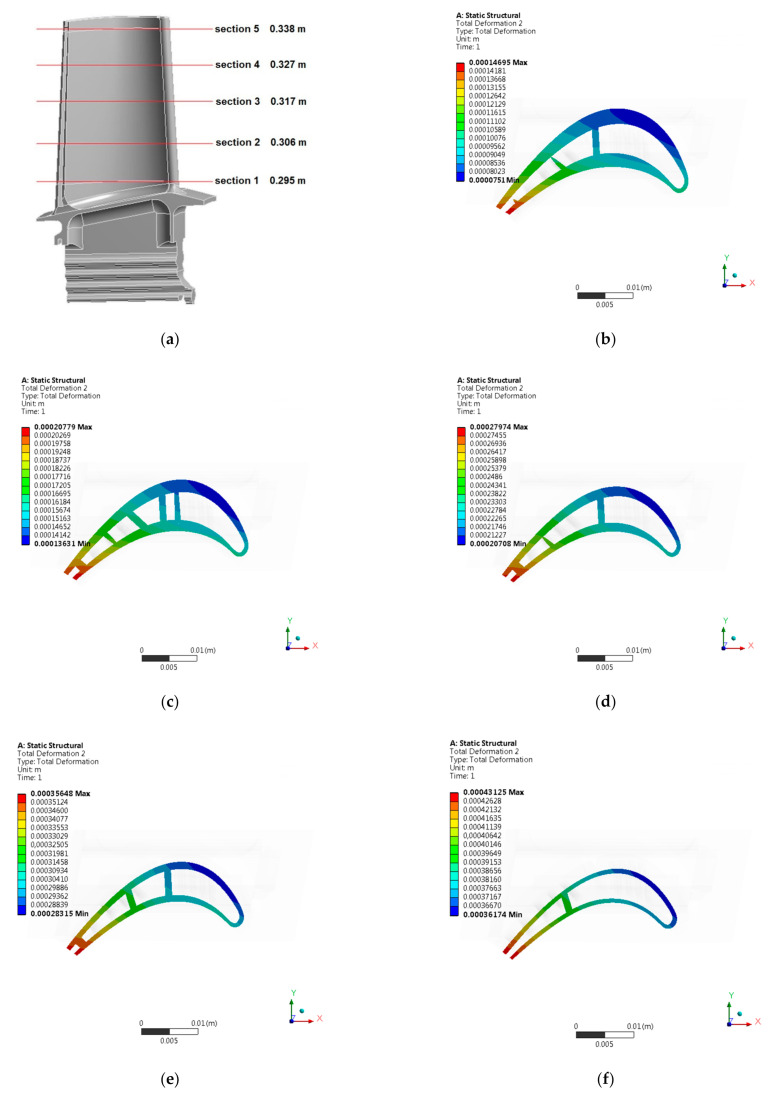
Position of the specific sections of the HPT blade (**a**); displacement [m] distribution in specific sections of the HPT blade: (**b**) Section 1; (**c**) Section 2; (**d**) Section 3; (**e**) Section 4; (**f**) Section 5.

**Figure 10:**
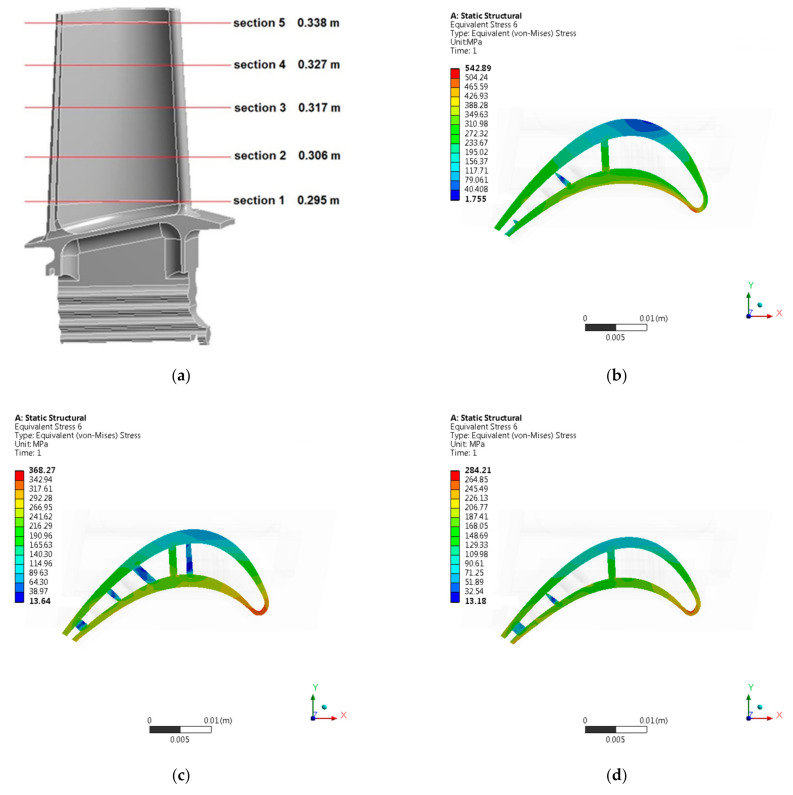

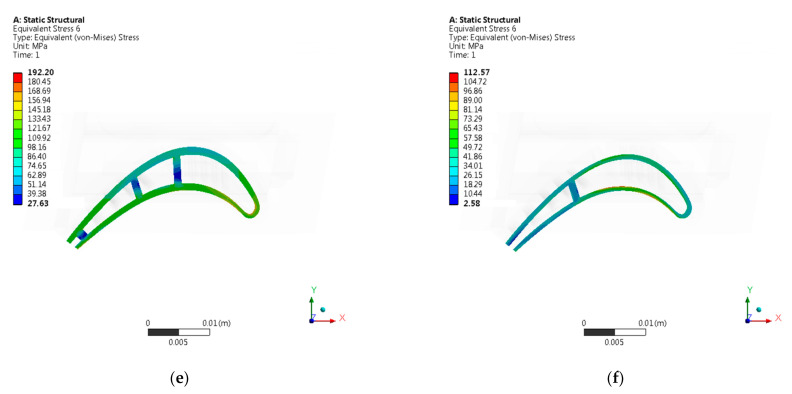
Position of the specific sections of the HPT blade (**a**); Equivalent stresses (von Mises) [MPa] distribution in specific sections of the HPT blade: (**b**) Section 1; (**c**) Section 2; (**d**) Section 3; (**e**) Section 4; (**f**) Section 5.

Figure 8 presents numerical analysis results in the form of a graphical distribution of displacements, equivalent stresses (von Mises), radial stresses, and equivalent stresses (von Mises) on a blade leading edge. The results are shown for the entire blade (Figure 8), as well as for selected sections (Figure 9 and Figure 10). The positions of specific sections were determined in relation to the lower surface of the blade attachment.

During performed numerical strength analysis, results were obtained which indicate that, in the area of the blade trailing edge, the greatest value of displacements occurs. Moreover, the value of displacements varies along the height of the blade, reaching the highest values at its top. On the other hand, the highest values of equivalent stresses (von Mises) were obtained in the area of the blade leading edge and its high-pressure side. The value of equivalent stresses (von Mises) changed along the height of the blade, reaching the highest values at its base. Table 2 shows the maximum values of displacements and stresses obtained for the entire blade and in specific sections.

To validate the numerical model, we decided to perform appropriate analytical calculations using strength of materials. To determine the value of stresses occurring in particular sections of the HPT blade along its length, the following formulas were used:

(1) $$\sigma_{n}=\frac{P_{n}}{F_{n}}$$

where:

$\sigma_{n}$—stresses in the *n*-th section of the blade [Pa],

$P_{n}$—force acting on the *n*-th cross-section of the blade [N],

$F_{n}$—area of the *n*-th cross-section of the blade [m^2^].

(2) $$P_{n}=\rho \omega^{2}\overset{i=n}{\underset{i=0}{\sum }}V_{i}\cdot {\bar{r}}_{i}^{\;}$$

where:

ρ—the density of the blade material [kg/m^3^],

ω—angular velocity of the blade/disc [rad/s],

${\bar{r}}_{i}^{\;}$—mean radius of the *i*-th segment of the blade [m],

$V_{i}$—volume of the *i*-th segment of the blade [m^3^].

**Figure 11:**
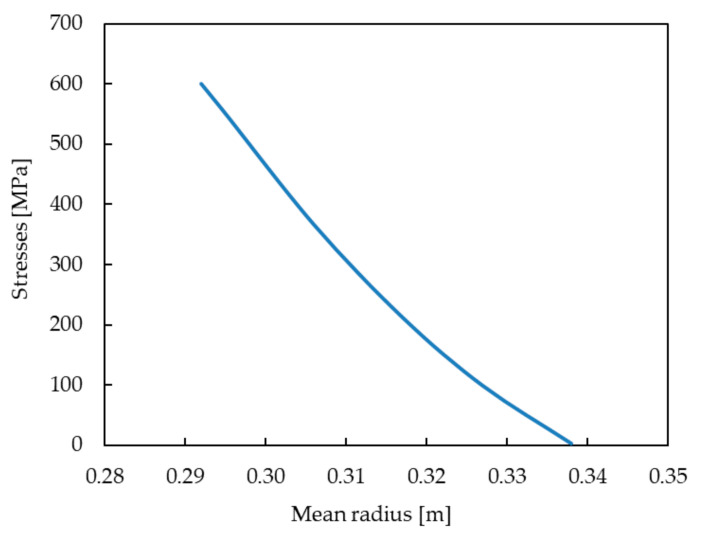
Change of stress values in the turbine blade.

During the calculations, the blade was divided into a selected number of segments. The volume of each segment was determined in Siemens NX software using the geometric model of the blade. Thanks to this, the volume of cooling channels inside the blade was taken into account and were subtracted from the analyzed material volume. Figure 11 shows the change of stress values in the turbine blade. The highest stress value obtained during the analytical calculations was 600.9 MPa and was found in the blade section near its base. It is worth noting that a high comparability of the analytical results with the results obtained from numerical analysis was achieved. This proves that the assumptions defined during the development of the HPT blade numerical model for the FEM analysis were correct.

### 3.4. High Temperature Fatigue Testing

**Figure 12:**
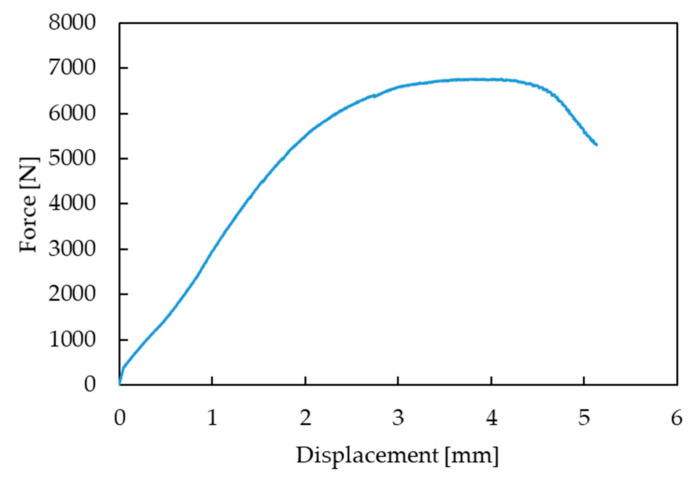
Static bending of the turbine blade at 950 °C.

**Figure 13:**
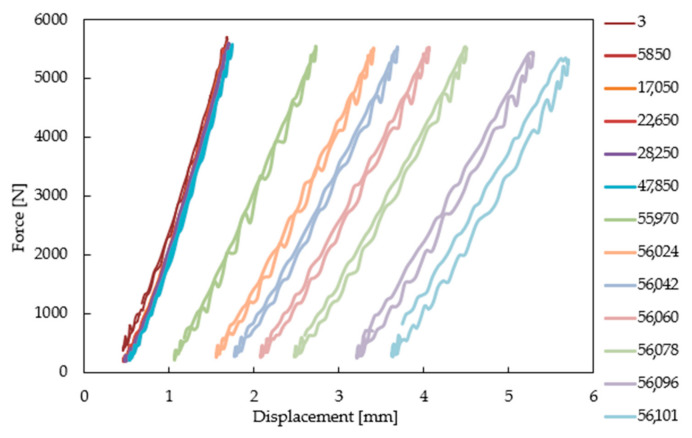
Selected hysteresis loops for the turbine blade subjected to cyclic loads of 5500 N.

**Figure 14:**
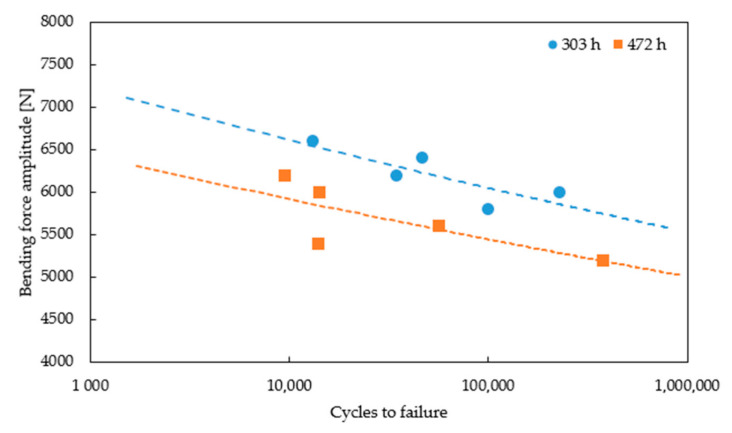
S-N curves for the turbine blades after different times of operation.

**Figure 15:**
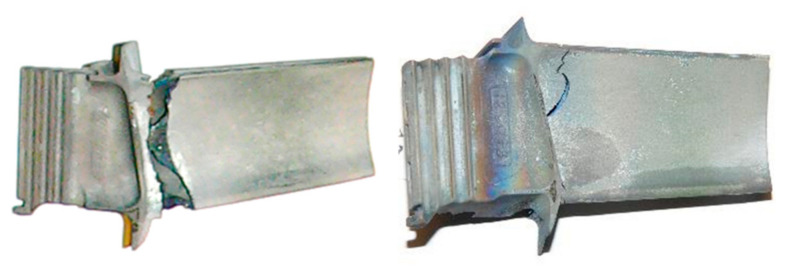
Photograph of the turbine blades after fatigue testing at 950 °C.

The range of bending forces used in high temperature fatigue tests was established on the basis of static bending of the turbine blade at 950 °C (Figure 12) using the experimental setup presented in Figure 3. Such tests allows us to determine the maximal force to failure. In the next step of the experimental program, fatigue comparative tests were performed to elaborate the S-N curves for both types of the turbine blades. Selected hysteresis loops for the turbine blade subjected to cyclic loads of 5500 N are presented in Figure 13. As it is clearly seen, due to a longer time of exploitation (472 h), a significant decrease of fatigue strength by a factor of 20% was observed for the entire range of stress amplitudes considered, as shown in Figure 14. The fracture analysis of turbine blades after high temperature tests performed indicate that the failure of turbine blades occurred near the grip area, at the bottom part of the blade (Figure 15). It should be highlighted that this particular area was found to be the region with the highest stress concertation predicted by the FE model. The correctness of the FE model was therefore confirmed by the experiment performed.

### 3.5. Microstructural Characterization of the Fractured Turbine Blade

**Figure 16:**
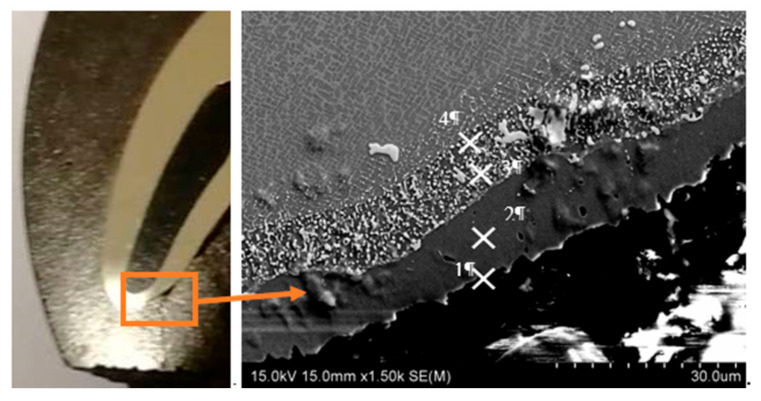
Photograph of the turbine blade metallographic section with SEM image after fatigue testing at 950 °C.

Microstructural characterization and chemical composition analysis was performed using scanning microscopy (Figure 16, Table 3). Microscopic observations revealed a typical structure of thermal barrier-coated nickel alloy with clear layer and interlayer zones. The coating itself consisted of two main sublayers. A chemical composition revealed that the outer sublayer, with a thickness of about 10 µm, contained mainly chromium (71.2% at) and nickel (21.6% at). In the interlayer area, aluminum (10.9% at.) was additionally observed. Such coatings effectively protect the substrate material against high temperature corrosion and oxidation [27].

## 4. Conclusions

The following conclusions were made in this study:

- The methodology and designed turbine blade grip could be successfully used to examine the high temperature fatigue performance of the full-scale turbine blade. The geometry of the rig itself allowed adoption of standard testing machines and thus simplified the complex high temperature testing of the turbine blades.
- The finite element model generated from the ANSYS software accurately predicted the area of potential failure of the turbine blade.
- Eddy current testing was successfully used to detect the most critical degraded region in the turbine blades, as the crack area accurately predicted by FE model was also localized as region A of the ET measurements, where the changes of phase angle were the most prominent.

## Figures and Tables

**Table 1:** Chemical composition of the turbine blades.

Element	C	Al	Si	Cr	Co	Ni	Nb
wt.%	2.1	6.2	4.4	4.1	10.0	70.8	2.4

**Table 2:** Results of numerical strength analysis of the high pressure turbine blade.

No	Name	Maximum Displacement [m]	Maximum Stresses [MPa]
1.	Entire HPT blade	0.445	598.8
2.	Section 1 of HPT blade	0.147	542.9
3.	Section 2 of HPT blade	0.208	368.3
4.	Section 3 of HPT blade	0.278	284.2
5.	Section 4 of HPT blade	0.357	192.2
6.	Section 5 of HPT blade	0.431	112.6

**Table 3:** Chemical analysis of the fractured area.

Point	C	Al	Si	Cr	Fe	Co	Ni
1	1.6	4.3	0.1	71.2	1.2	-	21.6
2	0.9	10.9	-	15.3	0.7	1.3	71.0
3	1.1	6.0	0.5	20.9	-	4.1	67.4
4	1.5	9.1	2.9	5.5	-	3.6	77.4

## Data Availability

Data available in a publicly accessible repository.
